# University of the Pacific—Medical Department

**Published:** 1859-03

**Authors:** 


### UNIVERSITY OF THE PACIFIC—MEDICAL DEPARTMENT.

We have received a circular, announcing the establishment of a Medical College in San Francisco, California.

The Regular Course of Lectures will commence on the first Monday in May, 1859, and be continued eighteen weeks. The following names are announced as the Faculty: J. Morrison, M. D., Prof. Pathology and Principles and Practice of Medicine; Isaac Rowell, M. D., Prof. of Chemistry; R. Beverly Cole, M. D., Prof. of Physiology, Obstetrics and the Diseases of Women and Children; E. S. Cooper, M. D., Prof. of Anatomy and Surgery; B. R. Carman, M. D., Prof. of Materia Medica, and Hon. Geo. Barstow, Prof. of Forensic Medicine.

We regard the establishment of a Medical College in California as a good move, and though we are not acquainted with the gentlemen composing the Faculty, we wish them the fullest success in their laudable effort to furnish Medical Education *at home.* We perceive however, that they have made one grand mistake in selecting the *summer* season for their lectures, without having first obtained permission from the University of Nashville, through the Convention of Colleges, and the American Medical Association.

